# Fever Nurses in Scotland

**Published:** 1920-11-20

**Authors:** 


### Fever Nurses in Scotland.

At a time when in England no precautions whatever were being taken to regulate the training of fever nurses, and when it was notorious that many women with mere experience in a fever hospital insinuated themselves through the medium of nursing institutes and doctors' references into the ranks of trained nurses in good practice, the Scottish Board of Health was taking precautions to ensure that fever training in that country should be a reality. A public examination was instituted for fever nurses, and in consequence the course of training was considerably levelled up. This fact, we think, fully explains the attitude of the Board of Health towards the General Nursing Council in Scotland, in urging the claims of the fever nurses qualified under their scheme to a place on the General Register.

Nevertheless times have changed, and the Board should realise that what was progressive ten years ago is less so nowadays. It cannot with any show of plausibility be argued that fever training, however well regulated, is the proper portal through which to enter the profession of nursing. It may be a useful preliminary to general training, as it is certainly an admirable supplement, but to open up a path whereby women specially trained in and for fever work should pose as thoroughly trained nurses on the General Register would lead to nothing but confusion and disappointment. The fever nurses' domain is a wide and lucrative one. She has no need to poach on that of her sisters trained for different duties. Both in private practice and in institutions for fever patients there is excellent and well paid work awaiting the fever nurses, and their claim to a separate Register of their own is questioned by few.

In the event of the Scottish Board of Health insisting on the admission of their fever-trained nurses being admitted to the General Register we foresee grave dislocation. For the English Board cannot possibly recognise this claim, and the result will at once be such a definite lowering of the qualifications required on the Scottish Register as must shatter reciprocity and introduce two standards into the United Kingdom. The issue is therefore a very grave one, and demands all the forbearance and wisdom available in nursing circles to bring about its solution.

